# Brain and Retinal Pericytes: Origin, Function and Role

**DOI:** 10.3389/fncel.2016.00020

**Published:** 2016-02-04

**Authors:** Andrea Trost, Simona Lange, Falk Schroedl, Daniela Bruckner, Karolina A. Motloch, Barbara Bogner, Alexandra Kaser-Eichberger, Clemens Strohmaier, Christian Runge, Ludwig Aigner, Francisco J. Rivera, Herbert A. Reitsamer

**Affiliations:** ^1^Research Program for Ophthalmology and Glaucoma Research, Paracelsus Medical University/SALK, University Clinic of Ophthalmology and OptometrySalzburg, Austria; ^2^Molecular Regenerative Medicine, Paracelsus Medical UniversitySalzburg, Austria; ^3^Spinal Cord Injury and Tissue Regeneration Center Salzburg, Paracelsus Medical University SalzburgSalzburg, Austria; ^4^Anatomy, Paracelsus Medical UniversitySalzburg, Austria

**Keywords:** pericytes, blood flow regulation, blood retina barrier, retinal diseases, tissue regeneration

## Abstract

Pericytes are specialized mural cells located at the abluminal surface of capillary blood vessels, embedded within the basement membrane. In the vascular network these multifunctional cells fulfil diverse functions, which are indispensable for proper homoeostasis. They serve as microvascular stabilizers, are potential regulators of microvascular blood flow and have a central role in angiogenesis, as they for example regulate endothelial cell proliferation. Furthermore, pericytes, as part of the neurovascular unit, are a major component of the blood-retina/brain barrier. CNS pericytes are a heterogenic cell population derived from mesodermal and neuro-ectodermal germ layers acting as modulators of stromal and niche environmental properties. In addition, they display multipotent differentiation potential making them an intriguing target for regenerative therapies. Pericyte-deficiencies can be cause or consequence of many kinds of diseases. In diabetes, for instance, pericyte-loss is a severe pathological process in diabetic retinopathy (DR) with detrimental consequences for eye sight in millions of patients. In this review, we provide an overview of our current understanding of CNS pericyte origin and function, with a special focus on the retina in the healthy and diseased. Finally, we highlight the role of pericytes in de- and regenerative processes.

## Introduction

Pericytes are specialized cells located at the abluminal surface of capillary blood vessels with key functions in vascular homoeostasis (Díaz-Flores et al., 2009). They were first described in the 1870’s by Eberth (1871) and Rouget (1879) and at that time named after the investigator “Rouget cells”. Due to their location in close proximity to endothelial cells, and their perivascular association to the microvasculature, Zimmermann (1923) later renamed them to “pericytes”. However, the definition and also proper identification of pericytes remains challenging. Depending on the vascular bed and their differentiation state, pericytes exhibit varying morphologies and express different marker profiles. In the last decades functional roles of pericytes in blood vessel stabilization (von Tell et al., 2006), in blood flow regulation (Hamilton et al., 2010), and in the formation of the blood-brain/retina barrier (BBB/BRB; Zlokovic, 2008; Bell et al., 2010; Winkler et al., 2011; Pfister et al., 2013) have been demonstrated. Further, pericytes possess a multipotent differentiation potential, which allows for generation of various different cell types (Crisan et al., 2009; Díaz-Flores et al., 2009). Due to this multipotency, these cells are a potential target for tissue repair and therapeutic approaches in regenerative medicine (Ozen et al., 2012).

This review covers pericytes within the central nervous system (CNS), focusing on retinal pericytes and their vascular functions as well as their contribution to retinal pathology progression. Because of its identical embryological origin, the mammalian retina is considered a part of the CNS. The nutrition of the retina is provided by two different vascular beds: the outer retina (photoreceptors) is passively supplied by the choroidal vasculature, whereas the inner retina is supplied by the retinal vasculature. The latter is comparable to CNS vasculature, constituting a tightly regulated cellular barrier. Retinal pericytes are essential constituents of the BRB and fulfill important functions to maintain vessel homeostasis. To provide a more comprehensive vision, this review will also include findings of other, non-retinal CNS pericytes. Finally, next to describing the vascular function of CNS pericytes the emerging hypothesis arguing in favor of a regenerative function and hence a therapeutic use of pericytes in tissue repair will be discussed.

## Origin of Pericytes

Pericytes are generated during embryonic and postnatal life (Armulik et al., 2011; Winkler et al., 2011). During developmental stages, CNS pericytes originate from neuroectodermal neural crest cells as demonstrated by quail-chick transplantation experiments of forebrain pericytes (Etchevers et al., 2001). These findings were supported by the possibility of neuroectodermal cells to differentiate into pericytes and vascular smooth muscle cells (vSMCs) of embryonic cerebral vessels (Korn et al., 2002). The neural crest origin of retinal pericytes was demonstrated using a Wnt-1 Cre-recombinase fate mapping mouse model, which specifically labels neural crest and neural crest–derived cells (Danielian et al., 1998; Gage et al., 2005). Within the retina, choroid and optic nerve, a neural crest origin was further demonstrated using a Sox10-Cre neural crest fate mapping mouse model (Trost et al., 2013). Along these lines, Simon et al. (2012) reported GFP-positive pericytes in the cortical gray matter using an inducible Sox10-Cre eGFP mouse model. Finally, neural crest origin of pericytes has also been described in thymic vessels of Wnt-1-Cre (Müller et al., 2008) and Sox10-Cre (Foster et al., 2008) mouse models.

In addition to the neural crest origin, pericytes potentially also derive from mesodermal cells (Etchevers et al., 2001). Using a XlacZ4 reporter under the control of an adipose tissue specific promoter (aP2), Tidhar et al. (2001) demonstrated reporter gene expression in vSMCs and pericytes throughout the vascular bed, including retinal microvessels. Also, bone marrow (BM) cells can be recruited during tumor- and cytokine-induced neoangiogenesis, giving rise to cells morphologically resembling pericytes and expressing the pericyte marker chondroitin sulfate proteoglycan 4/neural glial antigen 2 (NG2; Rajantie et al., 2004; Ziegelhoeffer et al., 2004; Song et al., 2005; Lamagna and Bergers, 2006). Finally, in corneal neovascularization almost half of the neovascular pericytes are BM-derived (Ozerdem et al., 2005). Also, fate mapping of GFP labeled BM using the stem cell antigen 1 promoter (sca-1), illustrated a BM origin of pericytes and a contribution of these cells to vascular remodeling during postnatal retinal angiogenesis as well as pathological angiogenesis in the retina (Pfister et al., 2013). Taken together, these data strongly suggest a mesodermal origin of pericytes resembling a pericyte reservoir for postnatal pathological neoangiogenesis.

## Identity of Pericytes

**Figure 1:**
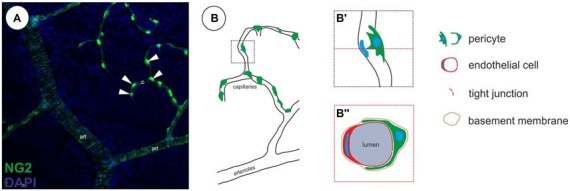
**Localization of pericytes within the retinal vasculature. (A)** Retinal vasculature, showing a single neural glial antigen 2 (NG2)-positive (green) vSMCs layer on arterioles (art) and single NG2-positive pericytes on capillaries (c, arrowheads; in accordance with results in Trost et al., 2015). **(B)** Illustration of the localization of pericytes on capillaries. **(B′)** Pericytes are located abluminal of endothelial cells, **(B″)** wrapping around the capillary, embedded within the same basement membrane as demonstrated in a capillary cross-section.

Under physiological conditions pericytes are located at the abluminal surface of microvessels, embedded in a common basement membrane with endothelial cells (Figure 1). The cytoplasmic processes of pericytes can span several endothelial cells and can have different morphologies (Dore-Duffy and Cleary, 2011), depending on the vascular bed and their differentiation/developmental state. The pericyte density and microvascular coverage varies according to the vascular bed, revealing the highest density in the CNS (Tilton et al., 1985; Frank et al., 1990).

**Figure 2:**
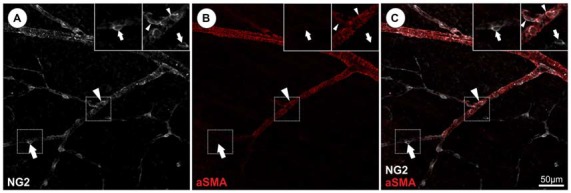
**Identification and discrimination of pericytes from vascular smooth muscle cells (vSMCs) in the retina visualizing NG2 and alpha smooth muscle actin (aSMA) protein expression. (A)** NG2 immunopositivity can be detected in both, retinal pericytes (representative arrow) and aSMA positive arteriolar SMCs (representative arrowhead). **(B)** Retinal capillary pericytes lack the aSMA signal (arrows) and **(C)** can be therefore discriminated from vSMCs as illustrated in the merged picture (in accordance with results in Trost et al., 2013).

Although the identification of pericytes by ultrastructural analysis may represent the “gold standard”, this method is not applicable in every experimental setting. Therefore a variety of potential pericyte-specific histological markers have been proposed (reviewed in Armulik et al., 2011), including platelet-derived growth factor receptor β (PDGFRb; Lindahl et al., 1997; Winkler et al., 2010), NG2 (Ozerdem et al., 2001; Trost et al., 2013), CD13 (Kunz et al., 1994), desmin (Nehls et al., 1992), vimentin (Bandopadhyay et al., 2001). In addition, the potassium channel complex Kir 6.1 has been used as a marker particular for CNS pericytes (Bondjers et al., 2006; Table 1). On the other hand, pericytes are negative for endothelial cell markers such as CD31 and von Willebrand factor and markers of other perivascular cell types, such as glial cells (GFAP, Olig2), microglial cells (Iba1) and neuronal cells. For the identification of retinal pericytes a combination of NG2 and PDGFRb can be recommended based on recently published data (Trost et al., 2013). However, it is important to note that the expression of antigens may differ for *in vitro* and *in vivo* conditions. For example, alpha smooth muscle actin (aSMA) has widely been used to identify pericytes and vSMCs *in vitro* and *in vivo*, however capillary pericytes do not express aSMA *in vivo* (Nehls and Drenckhahn, 1991; Trost et al., 2013; Hill et al., 2015). Therefore, aSMA labels vSMCs located on larger vessels, but not capillary pericytes. As several other markers such as NG2, PDGFRb or desmin are expressed by pericytes and vSMCS the identification and discrimination of these two cell types cannot be based solely on marker expression, but vascular localization must also be considered. As demonstrated in Figure 2, vSMCs on arterioles as well as pericytes on capillaries are labeled by NG2, however aSMA identifies only vSMCs on arterioles and is absent in retinal pericytes (Figure 2). This marker expression profile indicates a morphological and biochemical continuum from vSMC to pericytes, which is further supported by the common expression of the neural crest specific marker Sox10 (Trost et al., 2013). However, under pathological conditions pericytes may alter their expression. Therefore, the use of genetic models permanently labeling pericytes and their progenies (e.g., NG2-dsRed (Schallek et al., 2013) or NG2-CreERT2-eGFP mouse model (Hill et al., 2015) is essential and will enable to study the fate of pericytes also under pathological conditions). In summary, the specific identification of pericytes currently requires a combination of at least two markers as well as the consideration of the morphology and vascular localization and some circumstances will require appropriate transgenic animal models.

**Table 1:** **Summary of pericyte markers**.

Marker		Reference
PDGFRb	Platelet-derived growth factor receptor β	Lindahl et al. (1997) and Winkler et al. (2010)
NG2	Chondroitin sulfate proteoglycan 4/neural glial antigen 2	Ozerdem et al. (2001) and Trost et al. (2013)
CD13	Alanyl membrane aminopeptidase	Kunz et al. (1994)
Vimentin	Intermediate filament protein	Bandopadhyay et al. (2001)
Desmin	Desmin, structural protein	Nehls et al. (1992)
Kir6.1	Potassium inwardly rectifiying channel, subfamily J, member 8	Bondjers et al. (2006)

## Pericyte Functions

**Figure 3:**
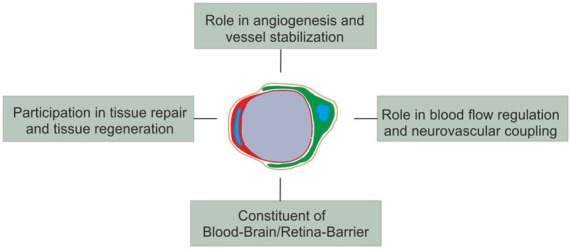
**Schematic overview of (the main) pericyte functions**.

Over the last decades different functions have been assigned to pericytes: they fulfill important functions during (a) angiogenesis and vessel stabilization; (b) participate in blood flow regulation and neurovascular coupling; and (c) are an essential constituent of the BBB/BRB. As mentioned above, next to their vascular functions they possess a multipotent differentiation potential (Figure 3).

### Angiogenesis and Pericyte-Endothelial Cell Interaction—The PDGF-B/PDGFRb Signaling Pathway

Pericytes play a major role in angiogenesis, participating in vessel formation, remodeling and stabilization (Gerhardt and Betsholtz, 2003). A number of signaling pathways and factors have been reported to be important for the intercellular communication between endothelial cells and pericytes, including transforming growth factor β (TGF β), angiopoietins, platelet-derived growth factor B (PDGF-B), spingosine-1-phosphate and Notch (reviewed in Armulik et al., 2005, 2011; Winkler et al., 2011).

Here, we will focus on PDGF-B/PDGFRb signaling, since this pathway is involved in pericyte proliferation, migration, survival and attachment. The importance of this signaling pathway was highlighted using genetically modified depletion and knockout models (reviewed in Betsholtz, 2004). During angiogenesis, sprouting endothelial cells secrete PDGF-B, which binds with high affinity to the pericyte-specific receptor PDGFRb, leading to the recruitment and attachment of pericytes. In general, impaired PDGF-B/PDGFRb signaling results in a failure of pericyte recruitment and in reduced microvascular pericyte coverage ultimately leading to endothelial hyperplasia, abnormal vascular morphogenesis and formation of microaneurysms (e.g., Lindahl et al., 1997; Hellström et al., 2001). Further, genetic deletion of either *pdgfrb* (Soriano, 1994) or *pdgfb* (Levéen et al., 1994) results in perinatal death due to vascular dysfunction. The mutation of particular downstream signal transduction molecule binding sites of PDGFRb resulted in a reduction of pericyte coverage in the heart and kidney vasculature, which was further decreased by crossing these mutants with PDGFRb null allele mice (Soriano, 1994). Investigating pericyte coverage in the retina in mice with multiple mutations, a reduced number of pericytes was detected and resulted in severe hemorrhage (Tallquist et al., 2003). The importance of PDGF-B is further underscored by studies demonstrating that ablation of endothelial derived PDGF-B resulted in reduced retinal pericyte coverage, leading to variable capillary and venous diameter, regressing capillary branches and the presence of microaneurysms. Furthermore, all mice showing <52% of normal pericyte density developed signs of proliferative retinopathy (Enge et al., 2002).

Another approach to investigate PDGF-B/PDGFRb signaling is the deletion of the retention motif in PDGF-B. The localization of the endothelial secreted PDGF-B in the vicinity of the developing vessel is guaranteed by binding of the PDGF-B retention motif to heparin sulfate proteoglycans (HSPG), resulting in subsequent binding of PDGFRb expressing pericytes to the endothelial tip cells (Lindblom et al., 2003; Armulik et al., 2005). Deleting this motif leads to impaired recruitment of pericytes, resulting in a severely disorganized retinal vasculature with partially detached pericytes and processes extending away from the vessel (Lindblom et al., 2003; Genové et al., 2014). As the truncated PDGF-B protein reveals full biological activity (Ostman et al., 1989), the endothelial localization of PDGF-B by the retention motif is suggested to be responsible for proper pericyte embedment in the microvessel wall. Besides the retention of PDGF-B at the developing vessel, the tissue concentration of PDGF-B is important for determining retinal pericyte density, as revealed by a reduction in pericyte coverage in mice with a monoallelic deficiency of PDGF-B (PDBF-B^+/−^; Hammes et al., 2002).

The impact of transgenic overexpression of PDGF ligands has also been investigated. Specific overexpression of PDGF-B in photoreceptor cells resulted in increased proliferation of pericytes, but also astrocytes and endothelial cells. These cells formed disorganized sheets and cords migrating into the inner retina, causing retinal tractions which ultimately resulted in a phenotype resembling retinal detachment in proliferative retinopathies. Furthermore, the formation of the deep capillary bed was inhibited in PDGF-B overexpressing mice (Seo et al., 2000; Mori et al., 2002; Vinores et al., 2003). The pathogenic effect of ectopic PDGF-B expression on retinal architecture and development was also demonstrated by overexpressing PDGF-B under the control of the myelin basic protein promoter (myelinating tracts), showing capillary and retinal disorganization (Forsberg-Nilsson et al., 2003). In line with this, expression of PDGF-B under the control of the nestin enhancer element (active in progenitor cells during development) resulted in severe retinal developmental defects, such as retinal folding, disorganized retinal lamination, delayed and abnormal vascular development, and progressive retinal degeneration (Edqvist et al., 2012).

It can be concluded that the balanced and well controlled secretion of endothelial PDGF-B, proper binding in close vicinity to the sprouting vessel and the function of the pericyte’s PDGFRb are essential for the proper development and maintenance of the CNS vasculature in general, and the retinal vasculature in particular.

### Blood Flow Regulation and Neurovascular Coupling

As pericytes express contractile proteins (Bandopadhyay et al., 2001) and are located on capillaries abluminal of endothelial cells, where vSMCs are absent, they are proposed to participate in microvascular blood flow regulation. Although the contractility and responsiveness of pericytes to vasoactive peptides has been demonstrated *in vitro* (Markhotina et al., 2007), concisive *in vivo* data remain limited. Evidence for pericyte contractility in response to vasoactive molecules/neurotransmitters was demonstrated using isolated retinal vessels (Kawamura et al., 2003; Wu et al., 2003) and by *in situ* studies using isolated rat retina (Schönfelder et al., 1998; Peppiatt et al., 2006) and cerebellar slices (Peppiatt et al., 2006). In line with these findings, Fernández-Klett et al. (2010) demonstrated the ability of pericytes to modulate local cerebral capillary blood flow in brain slice preparations *in situ* and cortex *in vivo*. In contrast, Hill et al. (2015) excluded a contribution of CNS pericytes to regulate cerebral blood flow on the capillary level. Focusing on aSMA-negative pericytes located on capillaries with a diameter of >10 μm, the authors detected a highly variable spontaneous vasomotion and calcium fluctuations, not correlating with changes in vessel diameter.

*In vivo* contractility of CNS pericytes and their influence on neurovascular coupling has been investigated in brain and to a lesser extent in retinal microvessels. The response of regional blood flow to neuronal activity (functional hyperemia) involves the interaction between neurons, glia and vascular cells (neurovascular coupling). Although Fernández-Klett et al. (2010) demonstrated active pericyte contraction after vasoconstrictor addition, they were unable to provide evidence that pericytes participate in neurovascular coupling after neuronal activity-induced hyperemia. In contrast, Hall et al. (2014) showed that cerebral pericytes actively dilate the capillaries after electrical whisker pad stimulation, where capillary dilation precedes arteriolar dilation. Although in both studies a similar experimental set-up was applied to monitor dilations of cerebral capillaries in response to neuronal activation, the mode of electrical stimulation to increase capillary blood flow was different and may account for the conflicting findings on the impact/ability of pericytes to increase cerebral blood flow.

Studying the impact of retinal pericytes on capillary blood flow regulation, the dilation of intermediate layer capillaries upon flicker stimuli was proposed to be mediated by active relaxation of pericytes, however without presenting direct evidence that pericytes actively dilated. Nevertheless, the authors concluded that functional hyperemia is driven primarily by active dilation of retinal arterioles, covered by vSMCs (Kornfield and Newman, 2014).

Although single studies propose a lack of pericyte contractility (Hill et al., 2015), currently the majority of studies suggests that CNS pericyte contractility is important to regulate local blood flow under pathological conditions, such as traumatic brain injury (Dore-Duffy et al., 2011) or ischemia (Peppiatt et al., 2006; Yemisci et al., 2009; Hall et al., 2014). Nevertheless, the active regulation of the capillary blood flow by pericytes remains a controversial issue, most likely due to the poor definition and identification of pericytes. Although several studies suggest pericytes to possess an important role in neurovascular coupling, more experimental *in vivo* evidence and a standardized definition of pericytes is necessary to compare and combine findings on the role of pericytes in blood flow regulation. Certainly, the use of genetically modified mice, labeling pericytes as well as vSMC in combination with pericytic markers will provide a more in depth and standardized tool to study the contribution of pericytes in blood flow regulation. Investigating pericytes in the retina for example, Schallek et al. (2013) imaged retinal pericytes non-invasively in the living eye using the NG2-dsRed mouse model. This model, also used to investigate cerebral blood flow (Hall et al., 2014), represents a great tool to study the impact of retinal pericytes on the regulation of capillary diameter.

### Blood-Brain-Barrier/Blood-Retina-Barrier and Associated Diseases

In the last 20 years, pericytes have been proven to be an essential constituent of the BBB and BRB. The BBB/BRB is a highly regulated barrier, controling paracellular flow between cells and transendothelial fluid transport, ensuring optimal chemical composition of the neuronal microenvironment and at the same time protecting from potential harmful substances. Although the main role in BBB/BRB tightness is mediated by tight and adherens junctions between endothelial cells, several studies have demonstrated that pericytes form and maintain together with endothelial, neuronal and glial cells the BBB/BRB and guarantee barrier function and tissue homeostasis. The BRB is composed of the inner BRB (retinal capillary endothelial cells) and the outer BRB (retinal pigment epithelial cells). Although the BBB is structurally similar to the inner BRB and both express several common transporters/receptors (Mori et al., 2003; Hosoya et al., 2009), some reports describe heterogeneous transport properties of the brain and retina neurovascular unit (André et al., 2012). The impact of pericyte loss to BBB and BRB breakdown and subsequent increased permeability has been reported in a multitude of studies: using PDGFRb signaling deficient mice (*Pdgfrb^+/+^, Pdgfrb^+/−^, Pdgfrb-F7*), Bell et al. (2010) demonstrated that reduced pericyte coverage in the brain results in BBB breakdown and an accumulation of plasma derived proteins, ultimately resulting in secondary neuronal degenerative alterations (e.g., impairments in learning and memory). Increased BBB permeability through pericyte deficiency was further demonstrated in studies using pericyte deficient mouse mutants (*Pdgfb^ret/ret^* and R26P^+/−^, R26P^+/0^) indicating a regulatory role of pericytes on the gene expression profile of endothelial cells and astrocytes (Armulik et al., 2010). The crucial role of pericytes in BBB formation was also confirmed during embryogenesis. Using different PDGFRb mouse mutants with reduced pericyte coverage (*Pdgfrb^−/−^*, *Pdgfrb^F7/F7^*, *Pdgfb^F7/−^*), Daneman et al. (2010) demonstrated a correlation of BBB permeability with pericyte loss, concluding that the pericyte amount determines the relative permeability of CNS vessels during development. The impact of pericytes on BBB mechanisms and subsequent neuronal damage has been investigated extensively using the aforementioned mouse models, however only few studies also investigated the impact of reduced pericyte coverage on BRB permeability and neuronal damage in the retina. Using endothelial specific PDGF-B transgenic mice (*Pdgf-b^ret/ret^*, *Pdgf-b^lox/−^*, *Pdgf-b^−/−^*), a loss of neuronal layers and folding of the photoreceptor layer in the retina was associated with reduced pericyte coverage, resembling signs of diabetic retinopathy (DR; Enge et al., 2002; Lindblom et al., 2003). Further, investigating tight junction formation in the developing retinal vasculature within the first three postnatal weeks, the contribution of pericytes to tight junction and BRB formation was demonstrated by the increased expression of the tight junction protein ZO-1 during maturation and enhanced pericyte coverage (Kim et al., 2009). However, a limitation of this study is the identification of pericytes using aSMA. As mentioned above, the development of tight junctions interconnecting endothelial cells is crucial for an intact BBB and the contribution of pericytes to tight junction formation at the BRB has been confirmed by electron microscopy *in vivo* as well as in endothelial-pericyte co-culture models (Daneman et al., 2010).

Taken together, as a disrupted BBB/BRB is associated with a variety of neuropathological processes, *in vitro* (Wisniewska-Kruk et al., 2012) as well as *in vivo* models are important to study the underlying mechanisms.

## Pericytes in Retinal Disease and Neuropathologies

Altered pericyte function and coverage have been described in diverse CNS diseases (reviewed in Lange et al., 2013). Diseases with a clear pericyte participation include DR (Beltramo and Porta, 2013), neonatal intraventricular hemorrhage (Braun et al., 2007), Alzheimer’s disease (AD; Winkler et al., 2014) or amyotrophic lateral sclerosis (ALS; Winkler et al., 2013) as well as diverse rare diseases like Cerebral Autosomal Dominant Arteriopathy with Subcortical Infarcts and Leukoencephalopathy (CADASIL; Table 2). One of the best studied pathological conditions associated with reduced pericyte coverage in the retina is DR. DR is a major complication of diabetes mellitus, characterized by increased vascular permeability (BRB breakdown), progressive vascular occlusion, microaneurysms and neuronal changes ultimately resulting in vision threatening diabetic macular edema and proliferative DR (Hammes et al., 2002, 2011). Although the pericyte loss is a well established fact of DR pathology, the underlying mechanisms for the development and progression of DR remain unclear (Frank, 2004; Qian and Ripps, 2011; Klaassen et al., 2013). Pericyte loss through apoptosis and destructive pathways under hyperglycemic conditions has been suggested (Behl et al., 2008, 2009). Altered glutamate excitation, reduced trophic factor signaling, oxidative stress, and neuroinflammation have also been associated with increased pericyte apoptosis (reviewed in Barber et al., 2011). Next to these alterations, growth factor-mediated pericyte depletion via angiopoietin-2/Tie-2 has been suggested (Hammes et al., 2004; Pfister et al., 2008; Park et al., 2014).

**Table 2:** **Excerpt of diseases with associated pericyte dysfunction**.

Disorders	Findings	Reference
Diabetic retinopathy	Reduced retinal pericyte coverage	Hammes et al. (2002, 2011), Beltramo and Porta (2013), and Klaassen et al. (2013)
Neonatal intraventricular hemorrhage	Decreased brain pericyte coverage	Braun et al. (2007)
Alzheimers disease	Degeneration of brain pericytes	Farkas et al. (2000), Sengillo et al. (2013), Sagare et al. (2013), Winkler et al. (2014), and Halliday et al. (2015)
Amyotrophic lateral sclerosis	Reduced pericyte coverage	Winkler et al. (2013)
CADASIL		Robinson et al. (2001), Haritoglou et al. (2004), and Roine et al. (2006)
Adams oliver syndrome	Reduced pericyte coverage	Patel et al. (2004)
Multiple sclerosis	Reduced brain pericyte amount	Kunz et al. (1995)
Stroke	Loss of capillary pericytes	Fernández-Klett et al. (2013)

Different genetically modified mouse models as well as pharmacologically induced diabetic rodent models (Enge et al., 2002; Huang et al., 2011; Lai and Lo, 2013; Qiu et al., 2015) have been used to study the molecular mechanisms of pericyte loss and vascular leakage in DR, but despite these available models, BRB breakdown remains poorly understood. Recently, Jadeja et al. (2013) characterized the causative mutation in the “redeye” mouse, identifying a mutation in the *PDGFRb* gene resulting in a reduction of normal/wt *PDGFRb* transcript. They verified this mouse being a useful model to study non-proliferative DR, demonstrating CNS-restricted, reduced pericyte coverage and BRB leakage followed by retinal neurodegeneration (i.e., reduced number of RGCs; Jadeja et al., 2013). To study the underlying molecular mechanisms at later stages of DR, the recently developed Akimba (Ins2^Akita^VEGF^+/−^) mouse, developing retinal leakage, neovascularization with hyperglycemia and signs of advanced clinical DR (diabetic macular edema, proliferative DR) might represent a suitable model (Wisniewska-Kruk et al., 2014).

The impact of pericytes as well as reduced pericyte coverage on microvessels is also discussed to contribute to glaucoma. While single studies report increased leakage of the BRB in the optic nerve head (ONH) in glaucomatous patients (Arend et al., 2005; Grieshaber and Flammer, 2007), the tightness of the BRB in this disease remains unclear. The density or degeneration of capillaries has been analyzed in several studies (May and Mittag, 2006; Mi et al., 2012; Almasieh et al., 2013), and vascular dysregulation has been suggested to be an important factor in the pathogenesis of glaucoma (Venkataraman et al., 2010). However, investigating pericyte coverage in an acute short-term glaucoma model revealed no alteration in pericyte coverage (Trost et al., 2015) and further studies are needed to understand the contribution of pericytes in the glaucomatous diseases.

Similarly to the retina, pericyte loss and dysfunction has also been reported to be associated with neurodegenerative disorders in other CNS regions. The degeneration of brain pericytes by specific amyloid-beta has been demonstrated by several studies *in vitro* (Verbeek et al., 2000; Rensink et al., 2002). In cerebral microvessels of patients suffering from AD, a degeneration of pericytes has been observed *in vivo* (Farkas et al., 2000) and this phenomenon was correlated with the severity of BBB breakdown (Sengillo et al., 2013). This finding was also confirmed in AD mouse models (Sagare et al., 2013; Halliday et al., 2015) further suggesting a contribution of pericytes to the pathogenesis of AD. Therefore, pericytes may represent a potential therapeutic target for AD (reviewed in Winkler et al., 2014).

Further, vSMC and pericyte degeneration has been described for the CADASIL syndrome caused by NOTCH 3 gene mutations (Dziewulska and Lewandowska, 2012; Gu et al., 2012; Craggs et al., 2015). It has been speculated that ischemic events and a consequently increased BBB permeability may account for the observed severe injury to the cerebral white matter in these patients. In addition, pericyte and vSMC degeneration has been confirmed in retinal vessels of CADASIL patients (Haritoglou et al., 2004), which is in accordance with observed alterations of the retinal vasculature (Robinson et al., 2001; Roine et al., 2006). In line with the above described findings, recently the aggregation of mutated Notch3 receptors on pericytes has been associated with the reduction of pericyte number and coverage of cerebral microvessels, resulting in the loss of BBB integrity and subsequent leakage of plasma proteins in a CADASIL mouse model (TgNotch3^R169C^; Ghosh et al., 2015). Another disease characterized by reduced pericyte coverage is the Adams-Oliver-Syndrome (AOS), associated with severe scalp and limb defects as well as impaired cardiovascular function (Patel et al., 2004). Furthermore, in an experimental mouse model for multiple sclerosis (MS), a reduction of pericytes has been detected and correlated to the functional state of the BBB (Kunz et al., 1995). As BBB dysregulation and transendothelial migration of activated leukocytes are among the earliest cerebrovascular abnormalities in MS (Ortiz et al., 2014), the contribution of pericytes to MS pathology is very likely. Finally, inducing cerebral ischemia in wild-type and pericyte specific reporter mice (*rgs5*^GFP^), as well as investigating histological samples of human stroke brains, Fernández-Klett et al. (2013) reported a progressive loss of capillary pericytes at the lesioned region.

Considering the fundamental role of pericytes in the formation and maintenance of the BRB/BBB and the impact of pericyte dysfunction pericytes represent a potential target for therapeutic therapies.

## Pericytes and Tissue Regeneration

Pericytes represent a very heterogeneous cell type displaying different embryonic origins ranging from mesodermal to neuroectodermal germ layers. Their capacity to differentiate into mesenchymal cell types (e.g., adipocytes, chondrocytes, osteoblasts, fibroblasts, vSMCs) has been proven in a multitude of studies (reviewed in Díaz-Flores et al., 2009), including CNS and especially retinal pericytes (Canfield et al., 1996; Doherty et al., 1998; Farrington-Rock et al., 2004). Furthermore, pericytes have the ability to differentiate also into non-mesenchymal cell types, such as neural cells. For example, a recent study has shown the differentiation of a skeletal muscle pericyte subtype into Tuj1-expressing neurons (Birbrair et al., 2013). Moreover, the ability of CNS pericytes to differentiate into neurons and glial cells has been demonstrated (Dore-Duffy et al., 2006; Paul et al., 2012) and CNS pericytes can be reprogrammed into neuronal cells by ectopic expression of the pro-neurogenic fate determinants Sox2 and Mash1 (Karow et al., 2012). A recent study demonstrated that CNS pericytes, isolated from ischemic brain regions, are able to differentiate into neural and vascular lineage cells, suggesting their contribution to neurogenesis and vasculogenesis at sites of brain injury (Nakagomi et al., 2015). Besides the neural differentiation potential of CNS pericytes, evidence for their ability to differentiate into stromal cells has also been provided in spinal cord injury (SCI) models *in vivo*. A specific pericyte subtype was demonstrated to give rise to cells forming fibrotic scar tissue after SCI and was further found to be crucial for wound closure as shown by specific depletion of this type A pericyte population (Göritz et al., 2011). Consistent with these findings, a recent study proposed an increase of PDGFRb+ pericytes in the injured spinal cord region, intermingled with GFAP+ astrocytes (Matsushita et al., 2015). Using a Nestin-GFP/NG2-dsRed fate mapping model, the type I pericytes (Nestin−/NG2+) have been shown to participate in scar formation after SCI (Birbrair et al., 2014), however as these cells lack PDGFRb expression in the fibrous scar they probably represent an additional pericyte subtype. Taken together, the proliferation of CNS derived PDGFRb+ stromal cells, identified as pericytes, has been described in SCI (Göritz et al., 2011; Matsushita et al., 2015) and cerebral ischemia (Fernández-Klett et al., 2013), indicating a crucial role of CNS pericytes in pathological fibrotic processes, being a potential target for future therapeutic strategies. Further the potential of CNS pericytes to differentiate into mesodermal as well as neuroectodermal cell types highlights their potential role in regenerative processes.

Due to their similarity to mesenchymal stem cells (MSCs), in terms of their expression profile and their differentiation potential as well as of their stromal capability, CNS pericytes have been considered to be the “CNS-resident MSCs” (Lange et al., 2013). Indeed, similarly to MSCs, CNS pericytes- release a plethora of bioactive molecules able to regulate proliferation and migration of CNS progenitor cells (Choe et al., 2014; Maki et al., 2015). This last property provides pericytes with the ability to regulate CNS endogenous progenitor/stem cell function during development and regeneration. Therefore, on the one hand via their stromal features pericytes could create a regenerative milieu by modulating/enhancing CNS-resident progenitor cells function and on the other hand a direct differentiation of pericytes into CNS reparative cells would be conceivable due to their enormous cellular plasticity.

Until now there are several studies showing a contribution of peripheral and CNS pericytes in peripheral tissue regeneration, but there is a lack of studies describing a regenerative capacity of CNS and especially, retinal pericytes in CNS tissue *in vivo*. In contrast, a multitude of studies report the capacity of pericytes from various peripheral tissues to improve and regenerate injured tissue: Crisan et al. (2008) isolated perivascular cells (CD146+/CD34−/CD45−/CD56−) from human skeletal muscle, pancreas, white adipose tissue and placenta, to demonstrate their myogenic potential and moreover the formation of myofibers after injection into the skeletal muscle of injured mice. The myogenic potential of human skeletal muscle pericytes was also confirmed in studies using a muscular dystrophy mouse model, showing host-muscle colonization and the generation of fibers expressing dystrophin (Dellavalle et al., 2007). As these cells, next to pericyte markers (NG2+, CD146+, aSMA+), also expressed markers typical for MSCs, the authors suggested that MSCs may have developed from perivascular cells/pericytes (Caplan, 2008; Crisan et al., 2008). The potential of MSCs to differentiate into distinct cell types such as bone, cartilage, tendon, fat or dermis and their participation in tissue regeneration has been described in a multitude of studies (reviewed in Caplan, 2007; Lange et al., 2013). The similarities between pericytes and MSCs highlight the relevance of pericytes in tissue regeneration. In addition to pericyte participation in myogenic regeneration, the potential to repair ischemic heart muscle has been provided in several recent studies: transplantation of skeletal muscle derived pericytes diminished ventricular dilatation and improved cardiac contractility in acutely infarcted mouse hearts (Chen et al., 2013). Avolio et al. (2015) provided evidence that cardiac pericytes possess the ability to penetrate and colonize xenograft tissues, which represents a potential future application in reconstructive surgery of congenital heart diseases. However, these cardiac “pericytes” were isolated by positive selection for CD34 and negative selection for CD146, which is in contrast used as a negative and positive selection marker respectively in the studies of Chen et al. (2013) and Avolio et al. (2015). This CD34+ population, located around the vasa vasorum in the adventitia of arteries and veins, expressed pericytic (NG2, PDGFRb, RGS5) as well as mesenchymal markers (CD44, CD90, CD73, CD29), probably representing a further pericyte subpopulation. Several studies report the regenerative capacity of this CD34+ pericyte population, as demonstrated in a mouse myocardial infarction model showing long term improvements of cardiac function after pericyte transplantation (Katare et al., 2011). These differences in pericyte isolation methods clearly indicate the necessity of standardized protocols to obtain homogenous and comparable pericyte preparations for use in translatable, regenerative therapeutic approaches. Furthermore, growing evidence suggests the existence of diverse pericyte subpopulations, fulfilling different functions, most likely also due to their varying anatomical locations. Two different pericyte subtypes have been identified by Birbrair et al. (2013), using a Nestin-GFP/NG2-dsRed transgenic mouse model. They investigated the contribution of pericyte subtypes to fibrotic tissue formation in models for diverse peripheral as well as CNS injuries and described a particular subtype, the “type I pericyte” (Nestin-GFP−/NG2-dsRed+), to accumulate near the site of injury (Birbrair et al., 2014). Furthermore, in an animal model with lung lesion, a fraction of pericytes was additionally shown to be involved in collagen production (Birbrair et al., 2014). The perivascular origin of collagen producing cells/myofibroblast has been further proposed for dermal scarring (Sundberg et al., 1996) and kidney fibrosis models (Humphreys et al., 2010).

Although, until now there is no study showing a positive effect of transplanted pericytes for any eye disease, there are some studies investigating regenerative properties of pericyte-related cells in eye pathologies. The first indication for a potential role of pericyte-like cells in regenerative processes in the retinal vasculopathies has been demonstrated by the use of adipose derived stem cells (ASCs). These cells represent an alternative type of adult MSCs expressing pericyte specific markers *in vitro* and further are able to differentiate into pericytes (NG2+/PDGFRb+/SMA+). The injection of ASCs after oxygen-induced retinopathy improved microvascular regrowth, and further prevented retinal capillary dropout in case of pre-injury injection, therefore providing proof for functional vascular protection (Mendel et al., 2013).

Although the differentiation potential of CNS pericytes, including retinal pericytes, has been demonstrated (Farrington-Rock et al., 2004; Dore-Duffy et al., 2006; Karow et al., 2012; Paul et al., 2012), to our knowledge no regenerative approaches have been conducted applying CNS pericytes in any kind of *in vivo* CNS (injury) models. As pericyte dysfunction and reduced pericyte coverage plays a crucial role in disease progression and as pericytes exhibit similar characteristics as MSCs, they seem to be highly suitable for cell-based therapies. Moreover, with increasing knowledge concerning CNS pericyte modulation and differentiation, future attempts may involve molecular therapies to modulate these cells *in vivo*.

## Conclusion

Pericytes play a crucial role in maintaining tissue homeostasis, and pericyte dysfunctions as well as a decrease in number underlie distinct pathologies. Their involvement in numerous diseases and their multipotency qualifies them as promising targets for future therapeutic regenerative approaches. CNS degenerative diseases and especially retinal degenerations often involve pathologies of the vasculature resulting in impaired vascular functions. As pericytes are crucially involved in vascular physiology as well as degenerative/regenerative processes, they are possible targets for therapeutic interventions. However, to fully harness pericytes as molecular “drug stores”, we need to increase our understanding of pericyte function in health and disease, leaving ample room for future studies.

## Author Contributions

AT, SL and FJR wrote the manuscript. FS, DB, KAM, BB, AK-E, CS, CR, LA and HAR critically reviewed the manuscript. All authors read and approved the final version of the manuscript.

## Conflict of Interest Statement

The authors declare that the research was conducted in the absence of any commercial or financial relationships that could be construed as a potential conflict of interest.

## Abbreviations

CNS: central nervous system
vSMC: vascular smooth muscle cells
GFP: green fluorescent protein
BM: bone marrow
BRB: blood retina barrier
BBB: blood brain barrier.
